# Epistemic disadvantage and looping breaks: a reply to Gauld et al.

**DOI:** 10.1007/s11019-026-10341-7

**Published:** 2026-03-06

**Authors:** Rena Alcalay

**Affiliations:** Department of Science, Technology and Society, Technical University of Munich, Munich, Germany

**Keywords:** Epistemic disadvantage, Epistemic injustice, Patient narratives, Nosological loop, Clinical exclusion

## Abstract

While Gauld et al. correctly argue that warranted clinical exclusion of patient narratives need not constitute epistemic injustice, this paper introduces epistemic disadvantage, a coextensive but distinct category of harm. Drawing on its three conditions, I show how epistemic harm persists even when exclusions are non-prejudicial and justified, contributing to clinical ethical obligations.

## Introduction

Gauld et al. (2025) argue in a recent article in this journal that not merely *any* clinical exclusion or disregard of a patient’s narrative constitutes epistemic injustice. They advance this position specifically in footnote 7, contending that when clinicians deliberately choose not to incorporate (all or part of) a patient’s account into their diagnostic reasoning, providing they have justifiable reasons for doing so and not on the basis of identity prejudice against the patient, then the failure should not be automatically categorized as “epistemically prejudicial and ethically harmful” (ft. 7).

Their argument hinges on an important distinction: they propose that epistemic injustice requires the presence of prejudices against the patient. According to their logic, clinicians may have legitimate clinical grounds for questioning or downplaying patient narratives, as they may be concerned that accepting a patient’s account verbatim could cause significant psychological distress, interfere with differential diagnosis, or indicate that the patient is being self-deceptive or dishonest. Additionally, they note that dismissing certain symptoms reported by a patient as non-integrative to diagnosis may not signal negative bias but rather reflect the heterogeneity of psychiatric disorders. In their view, questioning a patient’s narrative can be a protective clinical practice—a mechanism for avoiding diagnostic error. They illustrate this with an example of an adolescent caught in loyalty conflicts between divorced parents: the adolescent’s narrative, while meaningful, may not represent their objective mental health state and thus should not be taken at face value.

The core claim is that when a clinician’s disregard for the patient’s narrative is voluntary and based on sound clinical reasons, it should not be systematically considered epistemically prejudicial or ethically harmful.

The concept of epistemic disadvantage directly challenges this conclusion. Epistemic disadvantage, as I have developed it, offers a crucial corrective to Gauld et al.’s analysis by revealing a category of epistemic harm that can be coextensive with epistemic injustice—that is, ranging over related phenomena and highlighting different aspects, yet are not identical to them (Goldstein 2022; Alcalay 2024). Thus, epistemic harm encompasses a wider category than injustice alone.

## What is epistemic disadvantage?

Epistemic disadvantage occurs when warranted asymmetrical epistemic relations exclude a person or group from meaningful participation in knowledge exchanges, resulting in intellectual or moral harm. For example, when a medical practitioner prioritizes scientific evidence over a patient’s first-person experience, the patient may be harmed, yet the exclusion itself may be epistemically justified. Harm can occur within systems that are not fundamentally or ethically flawed, but where harm results from the way the system is utilized.

The key theoretical difference is that epistemic injustice requires unwarranted exclusions (often rooted in identity-based prejudicial attitudes), but not all epistemic harms are unjust, nor do all justified exclusions from knowledge exchanges avoid ethical harm. Epistemic disadvantage fills the conceptual gap by categorizing harms that arise from warranted, non-prejudicial asymmetrical relations where exclusion is based on proper evidential reasons (e.g., expertise differential, specialized knowledge requirements, or limitations in the system operation) and yet still the exclusion results in real intellectual or moral harm.

An instance in the history of anesthesiology helps illustrate this distinction. Early in the 21st century, experiments on child-patients using curare, a poison that causes paralysis but not numbing, led to outright dismissal of testimony that they could feel everything while in surgery. The exclusion of patient testimony indicated both an epistemic injustice and an epistemic disadvantage. Identity prejudice against children, rooted in the view that children lack full rational agency, exemplifies clear testimonial injustice. Yet the situation exposed nuances to epistemic harm: in reports and communications, physicians did acknowledge children’s testimony of pain but, lacking conceptual clarity, misattributed the pain to post-surgical disorientation rather than the absence of an anesthetic. Here, the exclusion did not stem from identity prejudice, but from a deficient application of epistemic resources. The resulting harm should be seen as an epistemic disadvantage arising from warranted exclusions. Although ethically harmful, it does not rise to the level of injustice.

Three features can help us recognize epistemic harms that are epistemically disadvantageous:

1. *The harm is not driven by identity prejudice* While Goldstein (2022) initially characterized epistemic disadvantage as involving non-deliberate harms, such harms need not be deliberate to constitute epistemic injustice (Spencer 2024: 224). The harm thus arises from warranted grounds like expertise differentials, specialized knowledge requirements, or justified clinical reasoning, rather than from prejudicial attitudes toward someone based on social identity. A clinician may have excellent clinical reasons for questioning a patient’s narrative without those reasons being rooted in stereotypes.
2. *A lack of conceptual resources to effectively communicate* the excluded party possesses first-person knowledge but lacks the technical vocabulary, conceptual sophistication, or epistemic precision to articulate their experience in ways that the epistemic community recognizes as epistemically relevant. A patient may know intimately that they are in pain yet lack the neurobiological framework to articulate where the anesthetic failed.
3. *The exclusion is warranted in the epistemic practice* epistemic disadvantage names harms that occur within a system that is not itself structurally unjust, but where harms result from the way the system is utilized. Medical expertise requires justified exclusion; the problem lies not in the existence of the exclusion but in how epistemic resources are understood and deployed within it.

Epistemic disadvantage yields intellectual and moral harms at both individual and systemic levels, often through first-order exclusions that bar intelligible rendering of diverse knowledge systems. Intellectually, individuals face constrained epistemic agency: their experiential knowledge fails to integrate into clinical reasoning, fostering bodily doubt and isolation from the shared epistemic space. Systemically, this scales to diagnostic frameworks that overlook heterogeneous experiences. Morally, individuals endure objectification—reduced to mere states of affairs from which information is extracted (Fricker 2007: 132; Medina 2012)—which can trigger physical stress symptoms, professional stagnation, or material losses. Systemically, harms like extractivist data withholding can limit collective intelligibility of varied knowledge practices (Alcalay and Vandekerckhove 2026).

### Why this matters for Gauld et al.’s argument

Gauld et al. argue that when clinicians deliberately disregard a patient’s narrative based on good clinical reasons, this “should not systematically be considered as epistemically prejudicial and ethically harmful” (ft. 7). But epistemic disadvantage suggests a more nuanced picture: the exclusion may indeed be warranted and non-prejudicial, yet the patient can still experience genuine epistemic harm.

A clinician questions a patient’s narrative for sound clinical reasons (e.g., concerns about psychological harm or interference with diagnosis), but the exclusion is not driven by identity prejudice (condition 1). The patient may lack the medical terminology to distinguish subjective experience from clinically relevant symptoms (condition 2). The patient’s exclusion from diagnostic decision-making on the basis that they lack medical expertise is justified (condition 3).

Epistemic disadvantage occurs when warranted exclusions, such as reliance on expertise and formal protocols, are exactly what clinicians are trained to regard as best practice, yet these practices harm individuals or systems of knowledge. This kind of harm can be easily overlooked and harder to identify in clinical practice particularly. When appearing as secondary harms, such as misfitting diagnoses or patient disengagement, it is typically coded as mere clinical error rather than an as epistemic and moral problem.

Indeed, this is consistent with recent scholarship on the differences between epistemic wrongs and harms (Dunne 2024; cf. Meluvor 2025) Recent scholarship from Dunne (2024) advances the distinction between epistemic harm and epistemic wrong. They note that the concept of harm is broad, encompassing any outcome in which an agent suffers damage (p. 4). Importantly, they clarify that not all harm constitutes wrongs. Wrongs have historically been classified as legal or moral. For example, kidnapping is wrong because it violates the legal right to be free from constraint and fails to respect the moral duty of care owed to others (p. 4). Yet as Dunne and Kotsonis highlight, the converse is not always true: one can be wronged without being harmed and harmed without being wronged.

When a patient’s experiential knowledge is not adequately integrated, the patient’s epistemic agency is constrained; the gap between what the patient knows and what the medical system recognizes as knowable persists. This is the harm of epistemic disadvantage. It does not fully constitute epistemic injustice because it does not involve prejudice or wrongful exclusion. It is harm that emerges from the legitimate structure and constraints of medical knowledge-making.

## Conclusion

Recognizing epistemic disadvantage as a distinct ethical harm from epistemic injustice reshapes how we assess clinical decision-making and patient harm. Gauld et al.’s broader insight is sound: the complex dynamics involved in the interactions between clinicians, patients, and diagnostic classifications create both opportunities and risks. The clinical loop involving the interaction between clinician and patient, and the nosological loop involving the interaction between clinical experts and diagnostic classifications, offer opportunities for constructive feedback, but they also pose risks of epistemic injustices, which can lead to looping breaks.

Yet the framework benefits from the addition of epistemic disadvantage as a category of harm. Within these loops—both clinical and nosological—warranted, non-prejudicial exclusions of patient narratives can still generate epistemic disadvantage. When a clinician prioritizes diagnostic protocols over a patient’s subjective experience, the exclusion may be justified and free of identity prejudice, yet the patient experiences real epistemic harm: their knowledge is not integrated, their epistemic agency is constrained, and the gap between what they know and what the medical system recognizes as knowable persists. This harm does not constitute injustice in the sense that Gauld et al. rightly emphasize. But it remains ethically significant.

Understanding epistemic disadvantage as distinct from injustice may, surprisingly, strengthen clinicians’ ethical obligations. If clinicians recognize that warranted exclusions of patient narratives avoid epistemic injustice yet still incur harm, responsibility shifts: they must develop practices to acknowledge and mitigate it, such as redesigning communication protocols to help patients translate experiential knowledge into clinically legible terms, creating space for collaborative meaning-making, or revisiting diagnostic decisions when new conceptual resources emerge.

Yet there is a limit to what any individual clinician can achieve. For patients misdiagnosed due to lacking the technical vocabulary or epistemic precision to articulate their experience, post-hoc apology does little to alleviate suffering. Epistemic disadvantage marks harms that are often not fully resolvable, as some harms are inherent to knowledge production itself (Alcalay, in development).

This invites the question *what medical systems owe patients in contexts of warranted epistemic exclusion?* The ethical task is to ensure that the inevitable costs of knowledge production are not consistently borne by the same populations. This may require revising how medicine is practiced. Gauld et al.‘s analysis of the nosological loop (the interaction between clinical experts and diagnostic classifications) opens crucial terrain here. If epistemic disadvantage arises in the clinical loop, it likely permeates the nosological loop too, where patients’ voices are almost entirely absent. At present, authority over medical narratives and diagnostic categories rests largely with clinicians and classification committees; epistemic disadvantage shows that this authority structure can be ethically problematic even when exercised conscientiously.

Should patient testimony and experiential knowledge inform nosological deliberations? This question is especially acute in psychiatric contexts, where clinical and nosological loops shape not only clinical decisions but the very categories through which mental illness becomes intelligible. Rethinking the place of first‑person experience in nosology will require fuller, empirically informed work on implementation than this conceptual commentary can offer. Still, the framework of epistemic disadvantage points toward structural possibilities, such as including patient representatives in nosological revision processes or hospital guideline committees, specifically tasked with bringing in forms of experiential knowledge that are currently structurally excluded; institutionalized feedback loops in which recurring reports of “not quite fitting the category” trigger review of diagnostic practices or criteria; and recognizing some domains of narrative authority as shared or negotiated (for example, granting patients provisional authority over how ambiguity in their own trajectories are interpreted) which clinicians then work to translate rather than override.

The framework developed here is especially apt for chronic care contexts, where clinicians have time for reflective testimonial practices and forward-looking responsibility (e.g., revisiting decisions as conceptual resources evolve.) It is less directly applicable to emergency clinical reasoning, where immediate, intuitive action often requires provisional exclusions of patient narratives with which patients may later disagree. Recognizing epistemic disadvantage thus extends Gauld et al.’s position, deepening ethical attentiveness to harms persisting even when injustice is absent.
